# The Role of General and Selective Task Instructions on Students’ Processing of Multiple Conflicting Documents

**DOI:** 10.3389/fpsyg.2019.01958

**Published:** 2019-09-03

**Authors:** Raquel Cerdán, Maria del Carmen Marín

**Affiliations:** ^1^Research Unit on Reading, ERI Lectura, University of Valencia, Valencia, Spain; ^2^Pureza de María-Grao School, Valencia, Spain

**Keywords:** comprehension, multiple documents, functional reading, task-oriented reading, on-line reading

## Abstract

This study was designed to test the role of general and selective task instructions when processing documents, which vary as regards trustworthiness and position toward a conflicting topic. With selective task instructions, we refer to concrete guidelines as how to read the texts and how to select appropriate documents and contents, in contrast to general task instructions. Sixty-one secondary school students were presented with four different conflicting documents in an electronic learning environment and were told to write an essay based on the information from the texts. Only half of the students were told to only use information from two out of the four texts to write their essay (i.e., selective condition). As predicted, students told to focus on specific documents and not use all of them for the assigned task (i.e., selective condition) better discriminated the quality of documents and type of information for the task.

Imagine that a group of adolescent students are given a set of texts on a current controversial topic (e.g., advantages and disadvantages of participating in social media). Having these documents available, they are asked to write an argumentative essay explaining the possible advantages and disadvantages of participating in social networks, as part of their class activities. We might encounter this second scenario. Another group of secondary school students is given the same set of texts, but they are instructed to use information only from the documents they deem relevant for the task and not from the rest of documents (general vs. selective task instructions) and perform their task only from that specific set of selected documents.

The above two cases reflect situations that students could face while reading on the Internet on conflicting topics, that is, (1) to solve tasks that require the selection of relevant content from a set of texts, (2) to complete the tasks under different types of instructions (i.e., from more general to more selective), and (3) to read and extract the information from texts varying in their levels of trustworthiness and position toward a topic. In these scenarios, specific competences associated with the use of advanced literacy skills would be essential, as described in recent models of functional reading, such as *RESOLV* (i.e., Britt et al., 2018).

Adolescents use the Internet to complete their school assignments, and for personal enjoyment (Leu and Maykel, 2016). The successful handling of complex sets of information would be an essential requirement for adolescent students dealing with complex documents and the Internet (Rouet and Potocki, 2018). However, when adolescents attempt to learn or solve a task *via* the Internet, it may be difficult to decide which information sources to trust and it becomes critical for young readers to employ effective strategies to deal with the documents on the web (Alexander and The Disciplined Reading and Learning Research Laboratory, 2012; Goldman and Scardamalia, 2013; Bråten et al., in press).

Thus, research on how specific instructions and task orientations might support this process becomes especially relevant, as we will discuss in this paper. Specifically, we are concerned with how different task directions might influence students’ critical evaluation of documents (see section “Critical Evaluation of Multiple Documents”). This would add to a growing body of research on how task instructions influence students’ reading of multiple documents in functional reading scenarios (i.e., Rouet and Potocki, 2018) and would have clear practical implications for teachers designing learning situations based on multiple documents. We will discuss these issues next.

## Critical Evaluation of Multiple Documents

Given the possibilities the Internet provides nowadays for accessing different and varied set of documents, students would need to develop the skills to access, integrate, and evaluate information critically (Rouet and Potocki, 2018). One first component of critical evaluation of multiple documents is the analysis of source features (i.e., Bråten et al., 2009; Stromso et al., 2010). Source features can include any metadata embedded within or provided outside the body of semantic information that informs on a text’s origin, context, and purpose (Barzilai and Strømsø, 2018). Source information should be jointly considered with content information (Rouet, 2006). Thus, young readers should be capable of identifying different documents varying regarding different parameters such as trustworthiness and level of adjustment to task. Readers would prioritize that content which is regarded reliable and fitting the expectations from the assigned task, whereas they would disregard content from non-reliable and not related-to-task sources.

Research has extensively focused on how adolescent students consider source information (i.e., Stromso et al., 2010; Strømsø et al., 2013; Bråten et al., 2018). How readers make document selections based on source information, content information, or both (McCrudden et al., 2016) has also been analyzed in the literature. According to this research, the analysis of source features is critical as it would enable readers to evaluate and interpret semantic content provided across different texts (Braasch and Graesser, in press), and to organize mental representations of what was read (Rouet et al., 2016; Braasch and Bråten, 2017; Saux et al., 2017).

Consequently, when reading multiple documents on the Internet for school purposes, it would be strategic to be selective and focus on task-related documents. However, the analysis of source information is scarce in secondary school students (i.e., Goldman and Scardamalia, 2013). In fact, adolescents would rarely attend to, evaluate, and use information about source features when reading on the Internet (Britt and Aglinskas, 2002; Walraven et al., 2009; Braasch et al., 2013). This is why empirical studies aiming to clarify task conditions that would promote critical evaluation of documents become especially relevant and with direct educational implications.

Consequently, specific interventions, such as which type of instructions are given for reading, may impact how students discriminate the trustworthiness of a document and its task-based relevance (Macedo-Rouet et al., 2013). Several theoretical models describe that competent readers initiate reading by interpreting goals, plans, and values for assigned or self-generated reading experiences (Brand-Gruwel et al., 2009; Goldman et al., 2010; Rouet and Britt, 2011; Leu et al., 2013). These interpretations would guide the location and selection of documents to read (Brand-Gruwel et al., 2009; Goldman et al., 2010; Rouet and Britt, 2011; Leu et al., 2013). Specific task directions might support the selection and use of multiple documents for completing school assignments, as we will discuss in this paper.

Recently, Pérez et al. (2018) conducted an intervention study to enhance teenagers’ capability to evaluate information quality, focusing on source reliability. Trained students increased the references made to reliable sources in a transfer task presenting contradictory information across texts. This finding indicates that, when specifically supported under specific task instructions or training programs, teenagers might critically evaluate information and make informed decisions regarding document relevance.

A second relevant component of critical evaluation of multiple documents relates to how multiple conflicting documents presenting opposing views are processed. Independent of the nature of the controversy or students’ previous positions, textual documents might present different and opposing views on a specific topic, as expressed by the authors of these documents. When readers access these, they might need to identify conflicting arguments and integrate them to form a coherent view (i.e., Perfetti et al., 1999). However, there is evidence that readers have difficulties in identifying the different arguments to construct an integrated view. Specifically, readers fail to integrate information from different positions of a controversial issue or topic into their mental models, leading to a one-sided representation of the controversy (Britt et al., 1999). In such situations, readers might have been affected by a *text-belief consistency effect* (Richter and Maier, 2017), by which information which aligns to readers’ previous beliefs, previous knowledge, or perspective might be processed with further detail and better remembered.

To explain the effects that beliefs have on the comprehension of controversial topics in multiple documents, Richter and Maier (2017) proposed the *Two-Step Model of Validation.* According to this model, in a first step of routine validation, readers encounter text-belief consistent information, which would affect memory and comprehension in terms of a text-belief consistency effect. In a second step, however, if readers engage in strategic and deliberate processing of inconsistent information, they could process with detail the information, which goes counter to their initial position or beliefs. In sum, engaging readers in active processing of the inconsistent information in step two would facilitate the construction of an integrated mental model from a set of controversial texts. Thus, if students engage in effortful, elaborative processing to understand the key differences among a set of controversial texts, this appears to produce a more balanced representation in long-term memory, as has been shown with adults (Wiley, 2005; Maier and Richter, 2013). Thus, we might enhance the processing of inconsistent information by instructing readers to process a set of texts under different task instructions which would promote active processing of the texts, as we will argue in the next section.

## Impact of Task Instructions on Students’ Processing of Multiple Conflicting Documents

Reading might be regarded as a goal-directed activity (McCrudden and Schraw, 2007), and different task directions could be presented in the context of school-based assignments to facilitate the students’ processing of a set of controversial texts. McCrudden and Schraw (2007) proposed a general descriptive model of goal-focusing processes in reading. Within this model, relevance instructions, which help to allocate the reading resources strategically, play an important role in learning from text and range from more specific to more general. They describe two types of relevance instructions. Specific relevance instructions highlight discrete text elements, whereas general relevance instructions would cover broad themes, purposes, or contexts for reading (prompts that ask readers to read for a general reason, such as writing a summary).

Similarly, the *Task-based Relevance and Content Extraction* (TRACE) model (Rouet, 2006) signals the importance of the processing of task instructions and how these would guide the strategic decisions students undertake to use a set of documents to solve a specific reading goal. Furthermore, the RESOLV model proposed by Britt et al. (2018; see also Rouet et al., 2017) deepens into the role of task instructions. Based on the task directions received, readers would construct a task model which would incorporate the plans to solve the reading assignment and would be updated throughout the reading task.

In multiple documents reading situations, different types of reading instructions and tasks have been shown to facilitate the identification of the different opposing views of a specific topic and the creation of an integrated mental model of documents (Wiley and Voss, 1999; Cerdán and Vidal-Abarca, 2008). For instance, in Maier and Richter (2016), undergraduates read controversial texts on the topic of health risks caused by cell phones. Participants either read the texts with the goal to write a summary or an argumentative essay. Reading times were collected. When *reading to summarize*, longer reading times were identified for belief-consistent information. When reading to write an *argumentative essay*, cognitive resources were allocated in a more balanced way and equal reading times were identified for belief-consistent and belief-inconsistent texts. Similarly, McCrudden and Sparks (2014) made high-school students read a dual-position text that contained arguments for and against widening a local tunnel. Two types of reading instructions were provided: *either to focus on evidence and reasons vs. no instruction*. Most participants were in favor of widening the tunnel. After reading the materials, their beliefs were weaker when reading under the evidence instruction. No changes in beliefs were observed for the other group.

In sum, there is emerging evidence in the literature that informs how readers’ processing of multiple conflicting documents under different task instructions influences students’ critical reading. However, more research is needed to explain how receiving different types of reading instruction affects students’ processing and attention to relevant document features and strategic reading decisions when reading from several documents. This is precisely the aim of this study and the main contribution of this paper to the continuously growing body of literature on multiple documents and advance literacy skills, in general. We will present the study next.

## The Current Study

In this paper, we will focus on the role of two types of task instructions (i.e., general and selective task instructions) when selecting and processing documents each of them varying as regards trustworthiness and content type (i.e., position toward a topic). With *selective task instructions*, we are referring to concrete guidelines that prompt readers to select appropriate documents and contents (i.e., please use information only from two documents which might help you accomplish an essay task), in contrast to *general task instructions* (i.e., presenting a set of documents students can refer to for completing an essay task). Past research has especially analyzed how students interpret task instructions (i.e., Llorens and Cerdán, 2012) and the impact of different types of instructions for reading single documents (i.e., Cerdán et al., 2009). However, the analysis of how varying instruction affects the selection and processing of multiple conflicting documents is novel.

Students were presented with four documents that contained different views on the same topic (i.e., participation in social networks). Each of the documents varied as regards level of *trustworthiness* (i.e., documents from trustworthy and untrustworthy sources) and position on the topic (in favor, opposed), so every text offered a unique combination of level of trustworthiness and position. Students were instructed to read the texts to write an argument answering the question: “*Would you recommend participating in social networks? Elaborate your answer using the information from the texts.*” To facilitate the selection of documents according to level of trustworthiness and content type, we asked half of the students to use information only from two of the four documents that were available (i.e., *selective task instruction*). This condition would provide students with more specific academic instructions for the task that would likely: (1) increase students’ consideration of the trustworthiness of sources and (2) facilitate the discrimination of the type of information presented in the texts.

We predicted that selective task instructions (i.e., to use information from only two documents from a wider set to write an argumentative essay) would increase students’ active engagement in the analysis of critical document features such as source dimensions and content type, in contrast to a more open general instruction (i.e., to read a set of documents to write an argumentative essay). This active processing of document features fostered by a selective task instruction might make students prioritize the processing of trustworthy documents and content showing an inconsistent view, that is, content showing a different position to that held initially by students (i.e., Cerdán and Vidal-Abarca, 2008; Richter and Maier, 2017), facilitating the construction of a balanced mental model from the documents in conflict.

## Materials and Methods

### Participants

Sixty-one high-school school students, with a mean age of 16 years (*M* = 16.67, SD = 0.68), participated in the study. They were of Caucasian origin and gender-balanced. They were selected from two classes of first year of secondary school, from an urban secondary school from a southern European country. The experiment was contextualized as part of their class in Spanish language, where training on document use and advanced reading skills becomes essential. Within each class, they were randomly assigned to a general (use information from all the documents to write the essay, *N* = 32) or selective condition (use information from only two documents to write the essay, *N* = 29). Participants assigned to the two conditions did not differ in their comprehension skills (*p* > 0.05), as measured by a standardized comprehension test (TEC, Martinez et al., 2008).

The study was approved by the school direction committee. In addition, parents provided informed written consent to participate in the study. This study was carried out following the recommendations of the Research Ethics Committee of the University from the first author. To preserve the privacy of participants, no personal data were required. Instead, code identifiers were used to gather the different learning products.

### Materials

#### Texts and Tasks

Four texts of approximately 300 words each on the topic of social networks were selected, presenting the positive and negative aspects of participating in social networks. The selected pool of texts was regarded as controversial, given the type of information and authors’ position on the selected topic, which varied across texts. Thus, independent of the researchers or students’ perspective or initial position toward the topic, the texts selected texts presented a controversy in itself, as they supported or not with divergent arguments the participation in social networks.

The texts uniquely varied according to two dimensions: *level of trustworthiness* (e.g., trustworthy vs. untrustworthy) and *position on the topic* (i.e., showing advantages vs. disadvantages). All texts were selected from original web sources but were adapted for research purposes. The first text was selected and adapted by the experimenters from a web site from the WHO (World Health Organization), and mainly showed a positive view, consistent with the participants’ perspective (Title: “*WHO clarifies the public the benefits of social networks*”). It was classified as trustworthy and advantages text. The second text, also from a trustworthy source, would present the opposite perspective (trustworthy and disadvantages text, *Ministry of Industry alerts of the risks of social networks*). The third text would come from an untrustworthy source (personal blog with no credentials, *Jonathans’ Blog*) and mainly listed a set of advantages when participating in social networks (*Jonathan explains the benefits of participating in social networks*). Finally, the fourth text also came from an untrustworthy source (i.e., *Lazy Corner*) and presented a negative view on the topic (*Lazy corner: main disadvantages and risks of social networks*).

The level of trustworthiness was independently rated by means of a *ranking task by expert teachers*. A pool of six secondary school teachers were given the title, source, and body of the four selected texts. They were asked to rank order the texts according to their estimated level of trustworthiness. The level of agreement in their rank orders was greater than 95%, and the two highest ranked texts were coded as trustworthy, and the other two as untrustworthy. Discrepancies were solved through discussion with the main authors of this article.

The texts also varied as regards position on the topic: two showing primarily advantages of participating in social networks, the other two highlighting the main risks and disadvantages. To determine this dimension, a content analysis was performed by the two authors of this paper. Any document including key words or self-disclosing statements (in title and body of text) signaling the authors’ position toward the issue of social networks (i.e., such as risks, benefits, advantages, disadvantages) would be classified either as an *advantages document* or as a *disadvantages document*. Each of the authors independently classified each of the four texts according to these parameters. Agreement was higher than 95%. Minor discrepancies were solved through discussion. Finally, in order to independently validate the classification of the textual materials regarding trustworthiness and position on the topic, the texts were further read and assessed by two independent researchers familiarized with multiple documents research. They were given the texts and were asked to classify each of them regarding the two dimensions. The level of agreement with the initial classification was above 98%, and only minor discrepancies were solved through discussion.

The task consisted in solving an open-ended question having the texts available by providing a recommendation for the participation or not in social networks, based on the students’ reading of the texts. Half of the students were told to use information from only two of the four documents to answer the question (i.e., *selective task instruction*), whereas the rest of the students would follow the general indication to read the four available texts and answer the task presented to them (i.e., *general task instruction*) Please note that the literal wording of the general instruction was as follows: *“Below you will find a list of four texts on the topic of social networks that you can access afterwards to complete your task. Please read the texts following the order you wish and answer the following question: Would you recommend participating in social networks? Elaborate your answer using the information from the texts.”* Only those students in the selective condition received this specific task instruction, in addition to the previous information: *“Please use information from only two of the four available documents to complete your task.”*

Both the reading of texts and the answering of the task were done using *Read&Answer* (Vidal-Abarca et al., 2011). This software allows to register on-line reading behavior in a controlled manner, while simultaneously embedding the tasks to be performed.

Participants were presented with an instructions page where they read the task according to the assigned condition (i.e., general, selective). The four documents were listed below in a google-like display (list of documents available, with references to source and content), and students were told to select and read the sources they considered relevant for their question, following the order they wished. Only participants in the selective condition were asked to use information from only two of the four available documents, even though they were allowed to access and read the whole set of texts.

#### Read&Answer

The four texts and the task were presented to the students using *Read&Answer*. This software presents the texts and the task on the computer screen and it registers the students’ on-line activity. *Read&Answer* has successfully been used in similar task-oriented reading scenarios (Cerdán and Vidal-Abarca, 2008; Cerdán et al., 2009; Vidal-Abarca et al., 2011).

*Read&Answer* presents readers with a screen showing the full text. All text but the unit currently selected by the reader are masked. Readers unmask a unit by clicking on it; when they unmask another unit, the first one is remasked. In the present experiment, information was divided in paragraphs. Specifically, every text was divided into four different paragraphs. Readers could access the task screen, including the wording of the task and a space to answer, from the text screen. It is possible to move from the question screen to the texts screen, and vice versa. Read&Answer permits the inclusion of more than one text, as we did in this experiment.

In this experiment, students first viewed a table of contents including the instructions for reading and for performing the task. In addition, students could view the list of texts in the form of a google-like list. This list of documents included content and trustworthiness cues, which should guide students’ decision to access and read specific documents. Through links included in the main screen, students could either go to the task screen or navigate across the four texts.

### Procedure

The experiment included two sessions of approximately 1 h each. They took place within the students’ regular class activity and thanks to the collaboration of the school teachers. On the first session, participants completed a comprehension test and were then assessed on their general position toward the topic of study. This was done on paper. By means of a general inquiry question (i.e., *what is your view about social networks?*), students were measured their general position toward the conflicting set of documents. Students had to respond by marking one of the following elements: *In favor/against/no opinion*. It should be noted that 95% of the students signaled the in favor option, whereas only 5% of the sample signaled no opinion. The second session was performed on the computer using *Read&Answer* (Vidal-Abarca et al., 2011).

Participants were presented with an instructions page where they read the task according to the assigned condition (i.e., general, selective). The four documents were listed in the form of a google-like display (list of documents available, with references to source and content type), and students were told to select and read the documents that better helped them solve their task. Students were placed no time limit, and were allowed to read the texts and perform the task at their will, having the documents available.

Students could access the texts and the task screen following the order they wished. Only participants in the selective condition were told to use information from only two of the four available documents when elaborating the answer. That is to say, they might read documents to discard information, but the content included in the answers should be extracted and elaborated from only two documents.

## Analysis and Results

### Impact of General vs. Selective Task Instructions on Students’ On-Line Reading Behavior in Multiple Documents Settings

We first analyzed a set of on-line measures that would represent students’ strategic reading when dealing with multiple documents, under the influence of different types of instructions and documents varying on level of trustworthiness and content type. In order to test these effects, several one-way ANOVAs were conducted, with independent factor type of instruction (*Selective vs. General*) and dependent variables, the list of behavioral indicators which appear below, which would consistently consider the type of text (trustworthy vs. untrustworthy) and the position on the topic (advantages vs. disadvantages). SPSS IBM statistics v24 was used as statistical package.

#### Time Reading Table of Contents and Links

We analyzed the time (in milliseconds) reading the different types of links included in the table of contents. These measures would reflect students’ analysis of the different texts in terms of relevance for the task’s goal and level of trustworthiness. They should differ depending on the general vs. selective task instruction. The following specific measures were analyzed: *Time reading advantages text links; time reading disadvantages text links, time reading trustworthy links, time reading untrustworthy links and total time reading all the links* (*see* Table 1). Significant results were found for: *time reading disadvantages links, time reading trustworthy links and total time reading all the links*.

**Table 1:** Means and standard deviations for table of contents reading times (in milliseconds).

	General		Selective	
	Mean	SD	Mean	SD
Time reading advantages text links	20873.47	15676.796	21004.43	14137.420
Time reading disadvantages text links	18251.69	17842.643	36520.89	21844.485
Time reading trustworthy links	19098.19	18474.410	34807.14	22546.299
Time reading untrustworthy links	20026.97	15422.863	22718.18	13935.021
Total time reading links	78250.31	53541.465	115050.64	57578.804

Regarding *time reading disadvantages links*, students in the selective condition read them for longer (*M* = 36520.89, SD = 21844.45) in contrast to those assigned to the general group (*M* = 18251.69, SD = 17842.64), *F*(1, 57) =13.35, *p* = 0.001, partial *η*^2^ = 0.19. This reflects the greater awareness of students instructed to be more selective in their decision to focus on information which was probably new for them and not supporting their initial positive view toward the benefits of social networks.

For the variable time reading trustworthy links, significant differences were found between the general (*M* = 19098.19, SD = 18474.41) and selective condition (*M* = 34807.14, SD = 22546.30). Students told to use information from only two documents to perform the writing task were more aware of the levels of trustworthiness that documents presented. Hence, awareness of sources was increased in the selective task instruction, as predicted, *F*(1, 57) = 8.74, *p* = 0.005, partial *η*^2^ = 0.13.

Finally, students in the selective task instruction dedicated longer time to reading all the links (*M* = 115050.64, SD = 57578.80), in contrast to the general instruction (*M* = 78250.31, SD = 53541.46), *F*(1, 57) = 6.74, *p* = 0.012, partial *η*^2^ = 0.107. This result reflects the greater processing effort in the table of contents for those in the selective group. Given they were told to make a decision on the relevance of documents for their task and use information from only two documents to elaborate their essay, they dedicated greater resources to reading the titles of documents, where information about type of content and trustworthiness of each of the texts could be found. The extent to which this prior analysis had an impact on actual reading times and on the task should be observed by taking into account other on-line and off-line measures. We will see measures related to the reading process (i.e., reading of documents) and to the elaboration of the essay task next.

#### Time Reading Texts

We analyzed the amount of time (in milliseconds) dedicated to reading the different texts, as an indicator of the processing effort in reading the documents. It should be taken into account that this measure included both the initial time reading the documents before the task, and the possible revisits during task completion. Students in the selective group were allowed to read all the documents, but use only information from two documents when writing the answer.

The following reading time measures were analyzed: *time reading advantages texts, time reading disadvantages texts, time reading trustworthy and untrustworthy texts, total time reading all texts* (see Table 2). Significant effects were found for: *time reading advantages and disadvantages texts.*

**Table 2:** Means and standard deviations for texts reading times (in milliseconds).

	General		Selective	
	Mean	SD	Mean	SD
Time reading advantages texts	213383.00	116265.38	158787.55	91155.09
Time reading disadvantages texts	207367.94	110969.76	276509.34	98021.30
Time reading trustworthy texts	208345.25	119801.12	244036.97	136393.65
Time reading untrustworthy texts	212405.69	116553.28	191259.93	140119.47
Total time reading the texts	420750.94	174312.87	435296.90	117949.54

Texts showing main advantages of participating in social networks were read for longer by students in the general instruction condition (*M* = 213383.00, SD = 116265.38), in contrast to students in the selective instruction group (*M* = 158787.55, SD = 91155.09), *F*(1, 57) = 3.98, *p* = 0.051, partial *η*^2^ = 0.065. The precise opposite pattern was found for the texts showing disadvantages, *F*(1, 57) = 6.45, *p* = 0.014, partial *η*^2^ = 0.102, for selective (*M* = 276509.34, SD = 98021.309) and general group (*M* = 207367.94, SD = 110969.77), respectively. These results show how the selective instruction seemed to induce a focusing effect on a type of information that goes counter to the initial positive view that students had, as measured in this experiment, toward the use of social networks, thus, showing the facilitative effect of instructions enhancing critical analysis of texts to focus on inconsistent information, as predicted by Richter and Maier (2017).

No significant differences were found across groups for the variable time reading all the documents. In order to test if the instruction to focus on two texts was followed by the selective group, we complementary considered the *mean number of texts accessed* in the experimental session. Significant differences were obtained between the general group (*M* = 2.72, SD = 0.92) and the selective condition (*M =* 1.83, SD *=* 0.38), *F*(1, 57) = 22.35, *p* < 0.05, partial *η*^2^ = 0.28. These two measures jointly considered show that participants generally dedicated a similar amount of time to reading all the documents, regardless of condition. However, differences seemed to appear as regards pattern of reading (i.e., prioritizing documents, as shown by the number of texts measure) and which information they decided to use in their task, as we will see in the next set of measures.

### Impact of General vs. Specific Task Instructions on Task Performance When Writing Based on Multiple Conflicting Documents

We analyzed the type of idea included in the task, by using a coding system similar to that successfully used in previous studies in the area of multiple documents based on the analyses of independent ideas (i.e., Cerdán and Vidal-Abarca, 2008; Linderholm et al., 2016). Two experimenters coded all the protocols, with an interrater agreement higher than 90%. Similar to the on-line behavioral measures, several one-way ANOVAs were conducted, with independent factor type of instruction (*Selective vs. General*) and dependent variables, the list of learning indicators which appear below. SPSS IBM statistics v24 was used as statistical package.

We focused on the following variables, relevant for our design (see Table 3): *ideas showing advantages; ideas showing disadvantages; ideas from trustworthy documents;* and *ideas from untrustworthy documents*. Specifically, we computed the absolute frequency with which these different ideas appeared in students’ essays. In addition, we counted the number of explicit references to sources (title and author information), mainly trustworthy and untrustworthy. This latter variable should reflect how students encoded the different documents and which ones they were prioritizing.

**Table 3:** Means and standard deviations for task measures (absolute frequencies).

	General		Selective	
	Mean	SD	Mean	SD
Ideas showing advantages	3.47	1.70	2.00	1.58
Ideas showing disadvantages	3.59	2.81	3.79	1.59
Ideas from trustworthy documents	3.28	2.69	3.66	2.67
Ideas from untrustworthy documents	3.78	2.77	2.14	2.56
References to sources	0.22	0.49	0.62	0.90

Significant effects were found for the variables *ideas showing advantages* and *ideas from untrustworthy documents*. The general condition included a higher number of ideas showing advantages (*M* = 3.47, SD = 1.70), in contrast to the selective group (*M* = 2.00, SD = 1.58), *F*(1, 57) = 11.57, *p* = 0.001, partial *η*^2^ = 0.169. In addition, the general group also included more ideas from untrustworthy documents (*M* = 3.78, SD = 2.78), differently to the selective group, (*M* = 2.14, SD = 2.56), *F*(1, 57) = 5.43, *p* = 0.023, partial *η*^2^ = 0.087. Finally, a greater number of explicit references to sources was found for the selective group (*M* = 0.62, SD = 0.90) in comparison to the general condition (*M* = 0.22, SD = 0.49), *F*(1, 57) = 4.69, *p* = 0.035, partial *η*^2^ = 0.076. The differences might be even larger, as the overall texts to cite in each condition (two for the selective vs. two for the general group) were not considered in the calculation of the dependent measure. In general, this latter result supports the initial prediction that students, when given concrete and specific task instructions, seem to be able to pay greater attention to the type of document they should use for a precise task, being able to better differentiate between more and less trustworthy documents.

## Discussion

We design this study to analyze the role of general and selective task instructions when selecting and reading documents that vary as regards trustworthiness and position on a topic. With specific task instructions, we refer to concrete guidelines as how to read the texts and how to select appropriate documents and contents, in contrast to general task instructions. How to provide appropriate orientations to students to facilitate performance and learning from texts seems particularly relevant, both empirically and in practical terms, as it might affect the instructors’ daily practices. Past research has especially analyzed how students interpret task instructions (i.e., Llorens and Cerdán, 2012) and the impact of different types of instructions for reading single documents (i.e., Cerdán et al., 2009). However, the analysis of how varying instructions affect the selection and processing of multiple conflicting documents in electronic learning environments is novel, in our view.

Students were presented with four different types of documents on the computer screen and were told to write an essay based on the information from the texts. Only half of the students were told to only use information from two out of the four texts to write their essay (i.e., selective condition), whereas this specific instruction was not provided to the general group. The texts dealt on the topic of social networks, and varied as regards trustworthiness (i.e., trustworthy and untrustworthy) and position on the topic (i.e., showing advantages or disadvantages). We predicted that a selective task instruction to read a set of conflicting documents would especially facilitate students’ attention and deeper processing of trustworthy documents, which should be apparent both when reading the documents and when writing the task assignments. Training programs enhancing critical reading strategies have successfully increased sourcing behaviors in adolescents (Pérez et al., 2018). In addition, we hoped that a selective task instruction to focus on a subset of given documents to perform a task would make students especially process information presenting a different view to that held initially by students. According to Richter and Maier (2017), task instructions that make students read information critically would help to overcome a text-belief consistency effect. We were also hoping that a selective instruction would make students especially select and process more deeply those texts showing the disadvantages toward social networks, a type of information that would complement the initial positive position held by students.

We analyzed students’ reading behavior in the table of contents page and when reading the documents (i.e., reading time measures). We also considered the type of ideas included in their tasks and the inclusion of explicit references to sources. On-line reading patterns supported our hypothesis that students with selective reading instructions dedicated greater resources to initial reading of table of contents, especially. In the analysis of reading times of the actual texts, these students showed also a tendency to focus on information which seemed to go counter to their initial positive view toward the topic. Students were presented with four different types of documents on the computer screen and were told to write an essay based focus on new and inconsistent information (Richter and Maier, 2017), which would show that students in this condition read in a more selective and critical manner. Finally, the complementary analysis of task products reinforced the prediction that providing selective task instructions helps students to write based on information from trustworthy documents and include relevant information. In this article, the main focus of analysis was the processes observed while reading on-line and registered through the tool *Read&Answer* (Vidal-Abarca et al., 2011). Similar to other research using the same tool and a similar methodological approach (i.e., Cerdán et al., 2011), our main goal was to identify a set of reading strategies under different experimental conditions. Complementary, task products were analyzed, which helped to further interpret the experimental manipulations. Future designs on the impact of task manipulations could focus specifically on the relationship between on-line measurements and their differential contribution to task and learning outcomes.

In sum, our results show that students who were indicated to use two of the four documents for the task were more selective when deciding to read only those documents which had two characteristics: (1) they were more trustworthy and (2) they were against their initial positive view toward participating in social networks. This was reflected in their reading behavior. In addition, these students also included more ideas in their answers from trustworthy documents, as well as they included more references to sources. These results suggest that providing specific indications to students to foster their reflection on what document might be appropriate for the task seems to be an effective means of increasing students’ discrimination of sources and enhances critical reading of multiple conflicting documents. This result is especially relevant nowadays, as secondary school students will encounter different types of documents, to be used for different purposes (Rouet and Potocki, 2018). The analysis of which type of instructions and strategies will help students be more critical in the process of selecting a concrete document for a specific purpose is certainly essential, especially when reading on-line.

This research has some limitations, which we acknowledge. First, we did not measure students’ perceived sense of difficulty with appropriate measures such as the *Cognitive Load questionnaire for Multiple Document Reading* (CL-MDR, Cerdan et al., 2018). The effects found in the selective condition might have been due to a reduction in Cognitive Load due to the fewer number of texts to focus on, and not to the specific instruction in itself. Future research might consider measuring students’ level of perceived effort by means of valid instruments. Second, we did not measure the extent to which students were capable of identifying and integrating the different arguments that were included in the documents in a final learning measure, which is a critical aspect in multiple documents learning. In this study, our focus was mainly the analysis of specific ideas which would be included in the open-response, based on the inspection of the documents (i.e., Cerdán and Vidal-Abarca, 2008). The availability of documents while composing the task allowed us to determine with significant levels of precision the origin of these ideas in terms of the type of document, which was the ultimate goal of this study. However, the analysis of integrative aspects of essay writing in studies based on multiple documents is truly a need and a challenge for future studies (Primor and Katzir, 2018) which we acknowledge in this paper.

Moreover, the extent to which the students viewed the selected topic as controversial was not measured initially with flexible measurements, nor the degree of change after the experiment. Finally, there might be individual differences in how instructions were understood, which we did not consider. In fact, the specific wording of the instructions might influence students’ response and learning behavior (i.e., how open or narrow the instruction is worded). Future research should help to clarify these issues, overcome some of the previous limitations, as well as focus on the effects of different types of instructions in open web-based environments. This way, the situations we test might resemble more those reading tasks which students might encounter when reading for academic purposes or for leisure.

## Ethics Statement

The study was approved by the school principal. Both the school and parents provided informed written consent to participate in the study. This study was carried out in accordance with the recommendations of the Research Ethics Committee of the University from the first author. No approval was required as per institutional as well as national guidelines and regulations. In order to guarantee the privacy of participants, no personal data were provided. Code identifiers were used in order to gather the different learning materials from the students.

## Author Contributions

RC is mainly responsible for designing and conducting the study. MM was involved in data analysis and interpretation of results.

### Conflict of Interest Statement

The authors declare that the research was conducted in the absence of any commercial or financial relationships that could be construed as a potential conflict of interest.
